# Impact of Socioeconomic Status on Cancer Incidence Risk, Cancer Staging, and Survival of Patients with Colorectal Cancer under Universal Health Insurance Coverage in Taiwan

**DOI:** 10.3390/ijerph182212164

**Published:** 2021-11-19

**Authors:** Wei-Yin Kuo, Han-Sheng Hsu, Pei-Tseng Kung, Wen-Chen Tsai

**Affiliations:** 1 Department of Health Services Administration, China Medical University, Taichung 406040, Taiwan; u100050853@cmu.edu.tw (W.-Y.K.); u104013401@cmu.edu.tw (H.-S.H.); 2 Department of Healthcare Administration, Asia University, Taichung 41354, Taiwan; ptkung@asia.edu.tw; 3 Department of Medical Research, China Medical University Hospital, China Medical University, Taichung 404332, Taiwan

**Keywords:** colorectal cancer, socioeconomic status, cancer stage, risk of mortality, survival analysis

## Abstract

This study examined the impact of socioeconomic status on colorectal cancer risk, staging, and survival under the National Health Insurance (NHI) system in Taiwan. Monthly salary and education level were used as measures of socioeconomic status to observe the risk of colorectal cancer among individuals aged 40 years or above in 2006–2015 and survival outcomes of patients with colorectal cancer until the end of 2016. Data from 286,792 individuals were used in this study. Individuals with a monthly salary ≤Q1 were at a significantly lower incidence risk of colorectal cancer than those with a monthly salary >Q3 (HR = 0.80, 95% CI = 0.74–0.85), while those with elementary or lower education were at a significantly higher risk than those with junior college, university, or higher education (HR = 1.18, 95% CI = 1.06–1.31). The results show that socioeconomic status had no significant impact on colorectal cancer stage at diagnosis. Although salary was not associated with their risk of mortality, patients with colorectal cancer who had elementary or lower education incurred a significantly higher risk of mortality than those who had junior college, university, or higher education (HR = 1.39, 95% CI = 1.07–1.77). Education level is a significant determinant of the incidence risk and survival in patients with colorectal cancer, but only income significantly impacts incidence risk.

## 1. Introduction

Cancer tops the list of 10 leading causes of death in Taiwan; among all cancers, colorectal cancer ranks at the top in terms of incidence and third in terms of mortality. In 2017, colorectal cancer reported an age-standardized incidence rate of 42.93 in every population of 100,000 [1] and an age-standardized mortality rate of 14.4 per 100,000 [2].

Most studies that measured socioeconomic status using individual data selected education level and income as variables of interest [3]. Due to the National Health Insurance (NHI) coverage in Taiwan, salary-related data used to calculate the premiums of enrollees could be obtained. Hence, Taiwanese studies on the impact of socioeconomic status on diseases often considered such data a proxy measure of the income status of the insured [4,5,6].

Although the impact of socioeconomic status on the incidence, staging, and survival of colorectal cancer has been well-documented in the literature, research has yet to derive a consistent conclusion regarding the numerous impacts due to international disparities in culture and healthcare systems. Although most studies have corroborated the finding that individuals with lower socioeconomic status are at higher risk of colorectal cancer [7,8,9,10,11], more likely to be diagnosed with late-stage colorectal cancer [8,12,13,14], and prone to lower survival outcomes [15,16,17,18,19,20,21,22,23,24], numerous studies in countries with universal health insurance coverage have indicated that socioeconomic status has no significant impact on the incidence [25,26] and stage of colorectal cancer at diagnosis [27,28].

Since its introduction in 1995, the NHI system in Taiwan has provided citizens with equal access to healthcare and preventive care services, regardless of their socioeconomic status. In theory, this measure should significantly reduce any inequalities arising from socioeconomic status. Taiwanese residents aged 50–74 years are also entitled to free biennial colorectal cancer screenings, and the medical expenses incurred during treatment for all cancer patients (including colorectal cancer patients) are covered by the National Health Insurance (NHI) of the Ministry of Health and Welfare (MOHW), Taiwan. NHI also boasts a coverage rate of 99.8% of the population in Taiwan [29]. Although there are few obstacles to seeking medical advice, the incidence and mortality rates of colorectal cancer remain high in Taiwan. Although many studies from several countries have focused on the impact of socioeconomic status on colorectal cancer, most of them employed regional data on socioeconomic status [7,14,17,18,30,31]. Few studies have incorporated individual factors of socioeconomic status, such as income and education level, in the context of NHI. Therefore, the current study explores the impact of socioeconomic status on colorectal cancer risk, staging, and survival under a universal health insurance system.

## 2. Materials and Methods

### 2.1. Study Design

A retrospective cohort study was adopted to select Taiwanese residents aged 40 years or above by the end of 2005 as participants and observe whether they developed primary colorectal cancer in 2006–2015. The survival outcomes of colorectal cancer patients were also observed until the end of 2016. Moreover, individuals’ socioeconomic status was represented based on their income and education level.

### 2.2. Data Sources

Data were obtained from the NHI Research Database from 2004 to 2016, along with the Taiwan Cancer Registry Database and Taiwan Cause of Death Statistics [32]. These databases were provided by the Health and Welfare Data Science Center under MOHW, and personally identifiable information had been de-identified prior to release. Hence, the data collected complied with personal data protection regulations.

### 2.3. Study Samples

Data from residents in Taiwan who had reached the age of 40 years by the end of 2005 were selected from the databases to observe whether they developed primary colorectal cancer (ICD-O-3:C18–C21) between 1 January 2006 and 31 December 2015. The exclusion criteria were as follows: individuals with missing data on monthly salary (*n* = 30,708) and those with a history of catastrophic illness or injury prior to 1 January 2006 (*n* = 22,170). During the subsequent analysis on cancer stage and survival, cases with unknown cancer stage (*n* = 856) and those with a histology code different from that of primary colorectal cancer (*n* = 1014) were excluded. The NHI designated a list of 30 types of major diseases as catastrophic illnesses or injuries, including cancer, stroke, end-stage renal disease, type 1 diabetes, hemophilia, systemic sclerosis, systemic lupus erythematosus, and so on.

**Figure 1:**
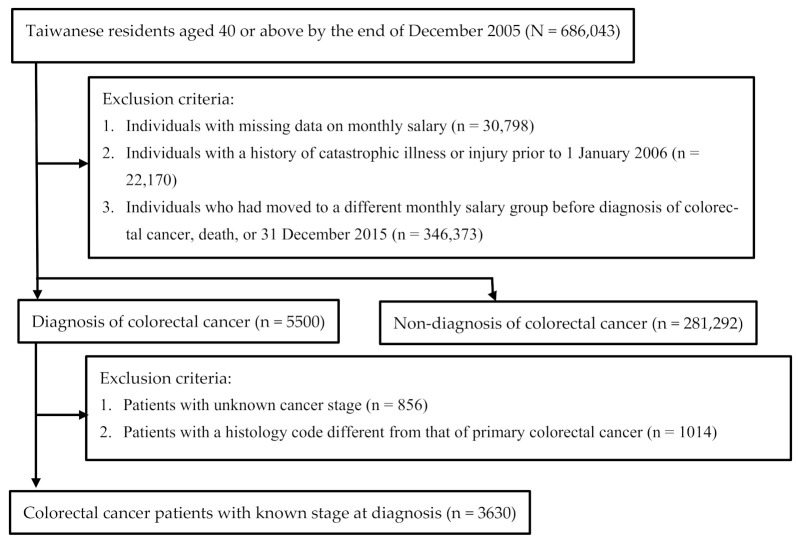
Selection process of study participants.

To mitigate the bias caused by dramatic fluctuations in salary, annual data on individuals’ monthly salaries were divided into three quartiles (≤25%, 25–75%, >75%), and individuals who had shifted to a different monthly salary group prior to diagnosis of colorectal cancer, death, or 31 December 2015 (*n* = 346,373) were excluded. The selection process of the study samples is outlined in Figure 1.

### 2.4. Measures

The dependent variables measured in this study included the incidence and stage of patients with colorectal cancer as well as their survival that measure the effectiveness of treatments [33]. The main independent variables were socioeconomic status (monthly salary and education level), whereas the control variables included demographic characteristics (gender, age, and marital status), health status (severity of comorbidity measured by the Charlson Comorbidity Index (CCI)), severity of illness (cancer stage), environmental factor (level of urbanization of the region of residence), and characteristics of the primary treating hospital (hospital ownership and level).

Socioeconomic status was measured based on individuals’ monthly salary and education level. Annual data on monthly salaries were sorted and then grouped into three quartiles (≤Q1, Q1–Q3, and >Q3). Grouping remained the same until diagnosis of colorectal cancer or the termination of observations. The sample was stratified into four groups based on education level—elementary school or lower education; junior high school; senior high or vocational school; and junior college, university, or higher education. The individuals in the study sample were aged 40 years or above; thus, their grouping should remain unchanged throughout the observation period.

Based on the demographic characteristics, the sample was classified into four groups by age (40–54 years, 55–64 years, 65–74 years, and ≥75 years) and three groups by marital status (single, married, and divorced or widowed). The severity of comorbidity was used as an indicator of health status and measured using the modified version of the CCI by Deyo et al. [34]. Based on the CCI scores calculated using medical records up to two years ago, the sample was divided into four groups (0, 1, 2, and ≥3). In terms of environmental factor, out of a total of 359 townships, cities, and districts across Taiwan, region of residence was assigned one of seven levels based on urbanization, with 1 being the highest and 7 the lowest [35]. The sample was then split into five groups—level 1, level 2, level 3, level 4, and levels 5–7. The treatment methods—referring to the treatments received by a colorectal cancer patient during the first year following diagnosis—were also divided into seven types: surgery; chemotherapy; radiotherapy; surgery and chemotherapy; surgery and radiotherapy; chemotherapy and radiotherapy; and a combination of surgery, chemotherapy, and radiotherapy. Finally, the primary treating hospital was defined as the hospital where a colorectal cancer patient had received the most cancer treatments during the first year following diagnosis; the most recent one prevailed if there were two or more hospitals with the same number of treatments. The hospitals were then divided into public and private based on ownership, while medical centers, regional hospitals, and district hospitals were divided based on the hospital level.

### 2.5. Statistical Analyses

All statistical analyses in this study were performed using the SAS statistical software package (Version 9.4; SAS Institute, Inc., Cary, NC, USA) [36]. A *p*-value less than 0.05 has been considered statistically significant. Descriptive statistics were adopted to describe the socioeconomic status and other control variables, and chi-square test was performed to identify any statistically significant difference (*p* < 0.05) in education level, gender, age, marital status, CCI, and urbanization level of the region of residence among individuals with different levels of monthly salary.

A log–rank test [37] was also used to identify any statistically significant difference in the incidence of colorectal cancer among residents with varying socioeconomic status. The Cox proportional hazards model [37] was applied to estimate the impact of socioeconomic status on the incidence risk of colorectal cancer when all other factors (such as demographic characteristics, health status, and environmental factor) were controlled for. Starting from the date of enrollment in the cohort study to 31 December 2015, participants who developed colorectal cancer during the observation period were considered as an event, and those who surrendered the insurance policy or did not develop colorectal cancer during the observation period were censored.

The chi-square test was also used to determine whether any significant differences were noted in the colorectal cancer stage at diagnosis among individuals with varying socioeconomic status. This study divided patients with colorectal cancer into early (stages I and II) and late (stages III and IV) stages and applied the logistic regression model to estimate the effects of varying socioeconomic status on the incidence of late-stage cancer (stages III and VI) when all other factors (such as demographic characteristics, health status, and environmental factor) were controlled.

The Cox proportional hazards model was used to investigate the influence of socioeconomic status of the relative risk of mortality in colorectal cancer patients with all other factors controlled for. Starting from the date of cancer diagnosis to 31 December 2016, participants who died from colorectal cancer during the observation period (classification of cause of death: C18–C21) were considered an event, whereas those who withdrew from the study, died from causes other than colorectal cancer, or did not die during the stated period were censored. The adjusted survival curves were plotted to illustrate the survival curves of patients with colorectal cancer with varying socioeconomic status when all other variables were controlled.

## 3. Results

### 3.1. Patient Characteristics

After eliminating samples based on the exclusion criteria, this study used data from 286,792 Taiwanese residents aged 40 years or above in 2006. In terms of socioeconomic status, Table 1 shows that 97,844 (34.13%) and 89,552 (31.23%) individuals had a monthly salary ≤Q1 and >Q3, respectively. In terms of education, 101,469 (35.38%) individuals with elementary or lower education accounted for the largest group, whereas 31,939 (11.14%) individuals had junior college, university, or higher education. Significant differences (*p* < 0.05) were observed in the education level, gender, age, marital status, severity of comorbidity, and urbanization level of the region where residents with varying monthly salaries are located.

### 3.2. Impact of Socioeconomic Status on the Incidence Risk of Colorectal Cancer

As shown in Table 2, a total of 5500 individuals (1.92%) in this study developed primary colorectal cancer for the first time between 1 January 2006 and 31 December 2015. The Cox proportional hazards model was used to analyze the impact of socioeconomic status on the participants’ relative risk of colorectal cancer when all other factors were controlled. After controlling for all other variables, residents with a monthly salary ≤Q1 had a 0.8-fold (95% CI: 0.74–0.85) increased risk of cancer compared with those who had a monthly salary >Q3, whereas those with a monthly salary between Q1 and Q3 were associated with a 1.11-fold (95% CI: 1.03–1.19) higher risk than those with a monthly salary >Q3. In terms of education level, after all other variables were controlled, residents with elementary or lower education and those who had completed junior high school faced a 1.18-fold (95% CI: 1.06–1.31) and 1.14-fold (95% CI: 1.02–1.28) increase the risk of cancer compared with individuals who had completed junior college, university, or higher education. No significant difference (*p* > 0.05) was noted in the risk of cancer between those with senior high or vocational school education and those with junior college, university, or higher education.

We further performed a Mantel–Haenszel chi-square test to examine interaction effects. We found an interaction relationship between age group and education level, as well as an interaction between age group and monthly salary on the incidence of colorectal cancer. Regardless of the age of 40–54, 55–64, or ≥65 years, people with monthly income ≤Q1 have a significantly lower risk of colorectal cancer than those with monthly income >Q3 (HR: 0.68–0.85, *p* < 0.05). For those aged between 40–54 and 55–64 years, the lower their education level, the higher their risk of colorectal cancer than those with college and above (HR: 1.12–1.35, *p* < 0.05). There was no significant correlation between education level and the risk of colorectal cancer for those aged ≥65 years (Table 3).

### 3.3. Impact of Socioeconomic Status on the Stage of Colorectal Cancer at Diagnosis

Table 4 presents the results of a logistic regression model used to examine the effects of varying socioeconomic status and other related factors on the stage of colorectal cancer at diagnosis. The findings revealed that, after all other factors were controlled for, salary and education level had no significant impact (*p* > 0.05) on the stage of colorectal cancer at diagnosis (i.e., early or late).

### 3.4. Impact of Socioeconomic Status on the Survival of Patients with Colorectal Cancer

**Figure 2:**
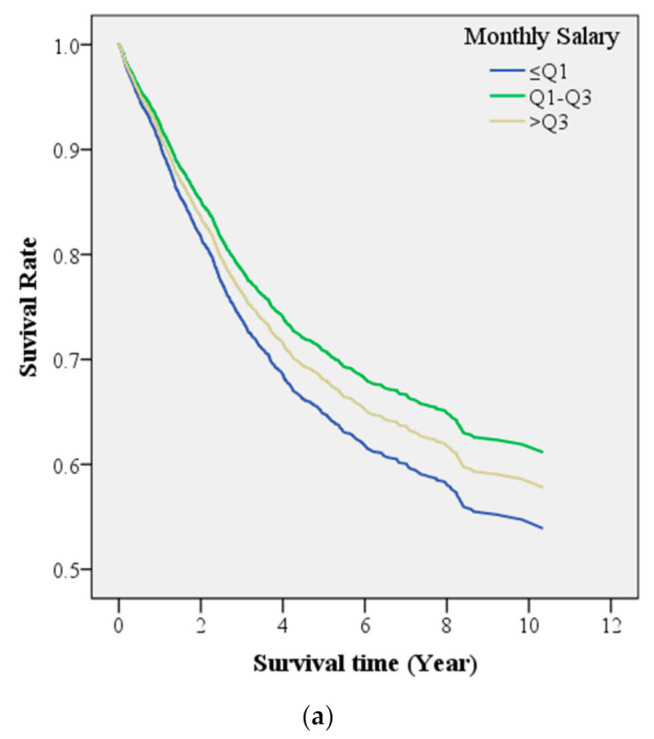

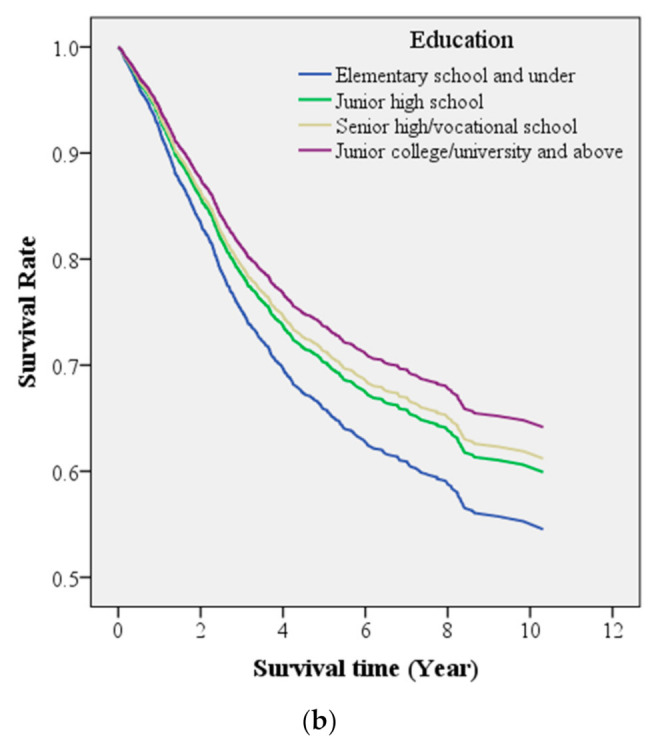
(**a**) The survival curves of colorectal cancer patients with varying monthly salaries. (**b**) The survival curves of colorectal cancer patients with varying education levels.

As shown in Table 4, the Cox proportional hazards model was adopted to analyze the impact of socioeconomic status on the relative risk of mortality in participants with colorectal cancer when all other factors were controlled for. The results show that salary had no significant influence (*p* > 0.05) on the risk of mortality in patients with colorectal cancer. Figure 2a illustrates the survival curves of colorectal cancer patients with varying monthly salaries. The survival rate was the lowest in patients with a monthly salary ≤Q1 and the highest in those with a monthly salary between Q1 and Q3. Regarding education level, patients with colorectal cancer who had elementary or lower education had a 1.39-fold (95% CI: 1.09–1.77) increased risk of mortality compared with those with junior college, university, or higher education. Patients who had completed junior high school and senior high or vocational school were at a slightly higher risk of mortality, but no significant difference was noted (*p* > 0.05). Figure 2b illustrates the survival curves of patients with colorectal cancer with varying education levels and shows that the survival rate increased with education level. Survival rate was the lowest in patients with elementary or lower education and the highest in those with junior college, university, or higher education.

Furthermore, this study stratified the sample based on monthly salary and analyzed the impact of education level and other variables on the survival of patients with colorectal cancer in each stratum. In the groups with monthly salaries ≤Q1 and between Q1 and Q3, education level had no significant impact on the risk of mortality in patients with colorectal cancer. However, within the group with monthly salaries >Q3, patients with elementary or lower education incurred a 1.96-fold (95% CI: 1.28–2.98) increased risk of mortality compared with those with junior college, university, or higher education (Table 5).

We further performed a Mantel–Haenszel chi-square test to examine interaction effects. We found an interaction relationship between age group and education level on the survival of colorectal cancer. This study used age stratification for further analysis. The results (Table 6) show that monthly income had no significant effect on the risk of death from colorectal cancer regardless of age group. Only among the 40–54-year-olds, those with an education level below junior high school level had a significantly higher risk of death than those with a university and above level (HR: 1.85–2.06, *p* < 0.05). There was no significant correlation between the risk of death and education level for people over 55 years of age (*p* > 0.05).

## 4. Discussion

The current study performed statistical analysis on large-scale national databases and used a total of 286,792 study samples to observe whether socioeconomic status affects the incidence risk and stage at diagnosis of colorectal cancer as well as the survival outcomes of patients. The results revealed that socioeconomic status impacts individuals’ risk of colorectal cancer and subsequent survival but exerts no significant influence on the cancer stage at diagnosis under a universal health insurance coverage (i.e., NHI). Education level is a key determinant; thus, individuals with lower education levels reported significantly higher incidence risk and lower survival outcomes.

After stratified analysis by age, this study found that the effect of education level on the incidence of colorectal cancer in different age groups mainly occurred in the population under 65 years. The lower the education level, the higher the risk of colorectal cancer.

A Finnish study focusing on this topic demonstrated that upper-level employees had significantly higher incidence risk of colorectal cancer compared with manual workers [38]. A US study also concluded that, in 2008–2011, higher income was associated with higher incidence rate of colorectal cancer compared with lower income [39]. Both findings are aligned with the current study results. Studies conducted in other countries with universal health insurance coverage (such as Sweden and Canada) found that income has no significant effect on the incidence of colorectal cancer [25,26]. However, the current study results show that individuals with a monthly salary ≤Q1 were at lower risk of colorectal cancer compared to those with a monthly salary >Q3 in Taiwan. A plausible explanation may be that higher-income individuals may face higher incidence risk because of their lifestyles. A study suggested that the higher incidence risk of colorectal cancer among individuals with high socioeconomic status may be attributed to their unhealthy lifestyles, characterized by, for example, insufficient physical activity, obesity, and overconsumption of red meat [40]. According to the results of the Nutrition and Health Survey in Taiwan, excessive daily intake of red meat was reported to be 24.3% among the Taiwanese population, whereas the prevalence of obesity (BMI ≥ 27) was high at 22.3% [41].

Studies in Sweden and the US indicated that the incidence rate was significantly higher in individuals with higher secondary or lower education compared to those with university or higher education [25,42]. In the case of Taiwan, the current study found that individuals with elementary or lower education and junior high school education were at significantly higher risk of colorectal cancer compared to those with junior college, university, or higher education. This may be because although the NHI system in Taiwan is effective in alleviating inequalities caused by economic factors, it is unable to effectively improve individuals’ health awareness, health behaviors, and other crucial determinants of colorectal cancer incidence. In this study, there was no statistically significant difference in the risk of colorectal cancer and the risk of death among people aged ≥65 years. However, in the population aged under 65 years, the lower the education level, the higher is the risk of morbidity and death from colorectal cancer. This study inferred that the current generations under 65 years in Taiwan generally have an education level of above high school. Therefore, if their education level is lower than junior high school, they may be poor in receiving health knowledge, health awareness, and health behaviors, which affect their cancer risk and death. For generations over 65 years old, their education level is generally junior high or elementary school, and the influence of education level was small, so the difference cannot be highlighted.

According to the aforementioned Swedish study, health awareness, which is highly influenced by education level, may be a key factor in affecting inequalities in the incidence of colorectal cancer [25].

Studies in Canada, the UK, and Australia showed no significant association between socioeconomic status and stage of colorectal cancer at diagnosis [27,28,43]. This finding is consistent with the current study results. However, other studies have shown that the proportion of late-stage diagnosis was higher in colorectal cancer patients with lower socioeconomic status [8,12,13,14,44]. The disparity may be explained using the NHI system in Taiwan. The Canadian study stated that the lack of a strong association between socioeconomic status and cancer stage at diagnosis may be because Canada has universal health coverage, which facilitates the implementation of cancer screening [27]. In addition to NHI coverage, Taiwan also has a free colorectal cancer screening policy in place, which makes screening available to any eligible individual who meets the age requirement. This has made the country effective in reducing inequalities associated with socioeconomic status. A Taiwanese study focusing on this topic stated that, after the policy was implemented, the proportion of stages II–IV colorectal cancer to all colorectal cancers decreased by 21% in the target population, with more than half of patients with colorectal cancer being diagnosed at stage 0–I [45]. This finding shows that the colorectal cancer screening policy significantly contributes to the prevention of advancing cancer stage.

Studies in the US and Canada found that patients with colorectal cancer who had lower household income were at significantly higher risk of mortality compared to those with higher household income [16,20,46,47]. This finding does not align with that of the current study. The reason may be two-fold. First, although the aforementioned studies exclusively used regional data on socioeconomic status, by measuring personal data on the individuals’ socioeconomic status, the current study could more accurately determine the characteristics of each participant. Second, as part of NHI policies, patients with cancer are entitled to exemptions of their medical expenses, which reduces the financial barrier for seeking medical advice faced by patients with colorectal cancer. High-risk groups are also offered free biennial colorectal cancer screenings to achieve the goal of early identification and treatment. A study showed that, under the influence of the colorectal cancer screening policy, the mortality risk of patients with colorectal cancer eligible for screening decreased by 7.49% in 2014–2017 compared to that in 1991–2003 [48]. This finding attested to the effectiveness of this policy in reducing disparities in the risk of mortality caused by economic factors.

Studies conducted in Sweden, Norway, and the US unanimously demonstrated that patients with colorectal cancer who had lower education levels experienced lower survival outcomes [20,22,23,24,49]. The current study also found that, in Taiwan, patients with colorectal cancer who had elementary or lower education were significantly likely to die compared to those with junior college, university, or higher education. A possible interpretation is that, although the policies surrounding the NHI system in Taiwan have rendered healthcare services highly accessible to patients with colorectal cancer, NHI policies have limited influence on certain factors, such as the treatment selection and patient adherence. According to a study conducted in the US, education is the key factor in dispelling any misconceptions between the physician and patient, and is highly important to individuals with lower socioeconomic status [50].

There were numerous limitations in this study. First, data on any significant association between variables, such as family medical history, lifestyle (diet and physical activity), and health behaviors (smoking and drinking) and colorectal cancer incidence or mortality cannot be obtained from the databases used in this study. Second, if any of the individuals had performed colorectal polypectomy previously as a result of a health examination, this may have lowered the incidence of colorectal cancer risk. However, this study was unable to obtain related data in this regard.

## 5. Conclusions

Socioeconomic status was associated with the risk of colorectal cancer, but the effects of monthly salary and education level varied. In terms of income, individuals with a monthly salary ≤Q1 were 20% less likely to develop colorectal cancer compared to those with a monthly salary >Q3, whereas individuals with a monthly salary between Q1 and Q3 were 11% more likely to do so. In terms of education level, individuals with elementary or lower education and junior high school education reported an 18% and 14% increase in the risk of colorectal cancer, respectively, compared to those with junior college, university, or higher education. However, both monthly salary and education level had no significant effect on the stage of colorectal cancer at diagnosis. Although monthly salary did not associate with the survival of patients with colorectal cancer, those with elementary or lower education had a 39% higher risk of mortality compared to those who had completed junior college, university, or higher education. Within the group with monthly salaries >Q3, the risk of mortality significantly increased by 96% in patients with elementary or lower education compared with those with junior college, university, or higher education.

After stratification by age group, there was no statistically significant difference in the risk of colorectal cancer and the risk of death among people aged ≥65 years. In the population aged under 65 years, the lower the education level, the higher is the risk of morbidity and death from colorectal cancer.

## Figures and Tables

**Table 1:** Demographic characteristics of study participants in 2006.

Variables	Total		Monthly Salary						
			≤Q1		Q1–Q3		>Q3		*p*-Value ^a^
	N	%	N	%	N	%	N	%	
Total	286,792	100	97,884	34.13	99,356	34.64	89,552	31.23	
Education									<0.001
Elementary school and under	101,469	35.38	43,081	42.46	17,406	17.15	40,982	40.39	
Junior high school	53,372	18.61	18,465	34.60	9150	17.14	25,757	48.26	
Senior high/vocational school	100,012	34.87	27,963	27.96	41,424	41.42	30,625	30.62	
Junior college/university and above	31,939	11.14	8375	26.22	1992	6.24	21,572	67.54	
Gender									<0.001
Male	152,943	53.33	53,033	34.68	44,496	29.09	55,414	36.23	
Female	133,849	46.67	44,851	33.51	54,860	40.99	34,138	25.50	
Age									
40–54	167,744	58.49	34,659	20.66	69,212	41.26	63,873	38.08	
55–64	47,864	16.69	19,778	41.32	17,066	35.66	11,020	23.02	
65–74	39,060	13.62	20,744	53.11	9077	23.24	9239	23.65	
≥75	32,124	11.20	22,703	70.67	4001	12.45	5420	16.87	
Marital status									<0.001
Single	25,407	8.86	14,515	57.13	5501	21.65	5391	21.22	
Married	221,416	77.20	62,696	28.32	82,369	37.20	76,351	34.48	
Divorced or widowed	39,969	13.94	20,673	51.72	11,486	28.74	7810	19.54	
CCI score									<0.001
0	169,420	59.07	49,755	29.37	62,963	37.16	56,702	33.47	
1	61,300	21.37	21,524	35.11	20,571	33.56	19,205	31.33	
2	27,091	9.45	11,720	43.26	8221	30.35	7150	26.39	
≥3	28,981	10.10	14,885	51.36	7601	26.23	6495	22.41	
Urbanization level									<0.001
Level 1	96,738	33.73	28,793	29.76	32,134	33.22	35,811	37.02	
Level 2	104,458	36.42	33,617	32.18	42,852	41.02	27,989	26.79	
Level 3	44,623	15.56	16,917	37.91	12,449	27.90	15,257	34.19	
Level 4	29,523	10.29	11,345	38.43	10,249	34.72	7929	26.86	
Levels 5–7	11,450	3.99	7212	62.99	1672	14.60	2566	22.41	

^a^ Chi-square test.

**Table 2:** Relative risks of suffering from colorectal cancer in study participants.

Variables	With Cancer		Without Cancer		*p*-Value ^a^	aHR ^b^	95% CI		*p*-Value ^c^
	N	%	N	%					
Total	5500	1.92	281,292	98.08					
Monthly salary					<0.001				
≤Q1	2030	2.07	95,854	97.93		0.80	0.74	0.85	<0.001
Q1–Q3	1890	1.90	97,466	98.10		1.11	1.03	1.19	0.005
>Q3	1580	1.76	87,972	98.24		1.00			
Education					<0.001				
Elementary school and under	2498	2.46	98,971	97.54		1.18	1.06	1.31	0.002
Junior high school	916	1.72	52,456	98.28		1.14	1.02	1.28	0.020
Senior high/vocational school	1584	1.58	98,428	98.42		1.10	0.99	1.21	0.076
Junior college/university and above	502	1.57	31,437	98.43		1.00			
Gender					<0.001				
Male	3284	2.15	149,659	97.85		1.46	1.38	1.55	<0.001
Female	2216	1.66	131,633	98.34		1.00			
Age					<0.001				
40–54	1896	1.13	165,848	98.87		1.00			
55–64	1280	2.67	46,584	97.33		2.56	2.37	2.77	<0.001
65–74	1335	3.42	37,725	96.58		3.62	3.33	3.93	<0.001
≥75	989	3.08	31,135	96.92		4.42	4.03	4.86	<0.001
Marital status					<0.001				
Single	342	1.35	25,065	98.65		1.00			
Married	4294	1.94	217,122	98.06		1.10	0.99	1.23	0.084
Divorced or widowed	864	2.16	39,105	97.84		1.15	1.01	1.31	0.032
CCI score					<0.001				
0	2791	1.65	166,629	98.35		1.00			
1	1305	2.13	59,995	97.87		1.05	0.98	1.12	0.169
2	670	2.47	26,421	97.53		1.09	1.00	1.19	0.048
≥3	734	2.53	28,247	97.47		1.14	1.04	1.24	0.003
Urbanization level					0.002				
Level 1	1933	2.00	94,805	98.00		1.00			
Level 2	2071	1.98	102,387	98.02		0.97	0.91	1.03	0.339
Level 3	799	1.79	43,824	98.21		0.91	0.84	0.99	0.032
Level 4	525	1.78	28,998	98.22		0.92	0.83	1.01	0.085
Levels 5–7	172	1.50	11,278	98.50		0.81	0.69	0.95	0.009

^a^ Log–rank test; ^b^ adjusted for monthly salary, education, gender, age, marital status, CCI score, urbanization level; ^c^ Cox proportional hazards model.

**Table 3:** Incidence risk of colorectal cancer in study participants stratified by age.

Variables	Age 40–54				Age 55–64				Age ≥ 65			
	aHR ^a^	95% CI		*p*-Value ^b^	aHR ^a^	95% CI		*p*-Value ^b^	aHR ^a^	95% CI		*p*-Value ^b^
Monthly salary												
≤Q1	0.85	0.79	0.91	<0.001	0.76	0.66	0.88	<0.001	0.68	0.61	0.75	<0.001
Q1–Q3	1.06	0.99	1.14	0.092	0.92	0.80	1.07	0.265	1.02	0.90	1.15	0.799
>Q3	1.00				1.00				1.00			
Education												
Elementary school and under	1.24	1.11	1.37	<0.001	1.23	1.00	1.53	0.051	0.89	0.75	1.06	0.177
Junior high school	1.19	1.06	1.33	0.002	1.35	1.06	1.72	0.014	1.00	0.83	1.22	0.981
Senior high/vocational school	1.12	1.01	1.24	0.025	1.29	1.04	1.59	0.022	1.02	0.85	1.22	0.869
Junior college/university and above	1.00				1.00				1.00			

^a^ Adjusted for gender, age, marital status, CCI score, urbanization level; ^b^ Cox proportional hazards model.

**Table 4:** Relative risks of suffering from colorectal cancer with late-stage and relevant factors.

Variables	Early Stage ^a^		Late Stage ^b^		*p*-Value ^c^	aOR ^d^	95% CI		*p*-Value ^e^
	N	%	N	%					
Total (*n* = 3630)	1576	43.42	2054	56.58					
Monthly salary					0.510				
≤Q1	591	43.52	767	56.48		1.01	0.85	1.20	0.930
Q1–Q3	534	42.28	729	57.72		1.05	0.88	1.26	0.565
>Q3	451	44.70	558	55.30		1.00			
Education					0.265				
Elementary school and under	717	43.19	943	56.81		1.14	0.88	1.48	0.314
Junior high school	242	40.81	351	59.19		1.24	0.94	1.65	0.133
Senior high/vocational school	466	44.00	593	56.00		1.10	0.86	1.43	0.445
Junior college/university and above	151	47.48	167	52.52		1.00			
Gender					0.164				
Male	956	44.36	1199	55.64		0.96	0.83	1.11	0.569
Female	620	42.03	855	57.97		1.00			
Age					0.867				
40–54	310	42.41	421	57.59		1.00			
55–64	417	42.99	553	57.01		1.01	0.83	1.24	0.909
65–74	373	43.68	481	56.32		1.00	0.80	1.25	0.985
≥75	476	44.28	599	55.72		0.97	0.77	1.21	0.763
Marital status					<0.001				
Single	81	35.68	146	64.32		1.00			
Married	1282	45.03	1565	54.97		0.68	0.51	0.91	0.009
Divorced or widowed	213	38.31	343	61.69		0.91	0.65	1.26	0.556
CCI score					0.069				
0	791	41.39	1120	58.61		1.00			
1	380	45.62	453	54.38		0.84	0.71	0.99	0.040
2	200	46.73	228	53.27		0.80	0.64	0.99	0.042
≥3	205	44.76	253	55.24		0.88	0.71	1.09	0.236
Urbanization level					0.866				
Level 1	563	43.24	739	56.76		1.00			
Level 2	586	43.63	757	56.37		0.98	0.84	1.15	0.836
Level 3	237	44.38	297	55.62		0.96	0.78	1.18	0.675
Level 4	143	43.33	187	56.67		0.99	0.77	1.26	0.929
Levels 5–7	47	38.84	74	61.16		1.25	0.85	1.84	0.259

^a^ Stage I and stage II; ^b^ stage III and stage IV; ^c^ chi-square test; ^d^ adjusted for monthly salary, education, gender, age, marital status, CCI score, urbanization level; ^e^ multivariable logistic regression.

**Table 5:** Impact of socioeconomic status on death risk of colorectal cancer patients.

Variables	All				≤Q1				Q1–Q3				>Q3			
	aHR ^a^	95% CI		*p*-Value ^b^	aHR ^c^	95% CI		*p*-Value ^b^	aHR ^c^	95% CI		*p*-Value ^b^	aHR ^c^	95% CI		*p*-Value ^b^
Monthly salary																
≤Q1	1.14	0.98	1.33	0.096												
Q1–Q3	0.87	0.75	1.03	0.099												
>Q3	1.00															
Education																
Elementary school and under	1.39	1.09	1.77	0.009	1.16	0.81	1.66	0.412	0.97	0.48	1.96	0.938	1.96	1.28	2.98	0.002
Junior high school	1.13	0.87	1.47	0.364	1.08	0.73	1.61	0.691	0.89	0.43	1.84	0.755	1.15	0.68	1.93	0.602
Senior high/vocational school	1.11	0.87	1.41	0.405	1.02	0.71	1.47	0.922	0.85	0.42	1.73	0.653	1.14	0.78	1.68	0.495
Junior college/university and above	1.00				1.00				1.00				1.00			
Gender																
Male	1.22	1.07	1.38	0.002	1.15	0.93	1.42	0.197	1.41	1.14	1.75	0.001	1.06	0.82	1.37	0.644
Female	1.00				1.00				1.00				1.00			
Age																
40–54	1.00				1.00				1.00				1.00			
55–64	0.84	0.70	1.01	0.058	1.05	0.71	1.56	0.805	0.73	0.55	0.96	0.027	0.93	0.67	1.31	0.684
65–74	1.06	0.87	1.29	0.566	1.11	0.76	1.63	0.582	1.29	0.93	1.77	0.124	0.97	0.67	1.41	0.869
≥75	1.47	1.20	1.78	<0.001	1.67	1.16	2.40	0.006	1.34	0.92	1.95	0.130	1.53	1.03	2.27	0.034
Marital status																
Single	1.00				1.00				1.00				1.00			
Married	0.97	0.76	1.24	0.824	0.99	0.71	1.37	0.943	0.77	0.48	1.26	0.302	0.75	0.36	1.57	0.446
Divorced or widowed	1.06	0.80	1.39	0.692	1.15	0.80	1.65	0.460	0.73	0.42	1.28	0.271	0.71	0.32	1.60	0.413
CCI score																
0	1.00				1.00				1.00				1.00			
1	0.94	0.80	1.09	0.391	1.17	0.92	1.48	0.192	1.04	0.79	1.36	0.778	0.61	0.45	0.84	0.002
2	1.09	0.90	1.31	0.393	1.21	0.91	1.60	0.194	0.88	0.60	1.29	0.508	1.04	0.71	1.52	0.838
≥3	1.26	1.05	1.50	0.013	1.38	1.06	1.80	0.018	1.06	0.74	1.53	0.737	1.40	0.97	2.01	0.070
Urbanization level																
Level 1	1.00				1.00				1.00				1.00			
Level 2	1.02	0.89	1.17	0.755	0.82	0.65	1.03	0.081	1.22	0.96	1.55	0.102	1.19	0.90	1.56	0.224
Level 3	1.01	0.84	1.21	0.932	0.99	0.74	1.31	0.917	0.94	0.66	1.34	0.731	1.27	0.89	1.81	0.190
Level 4	1.06	0.85	1.32	0.600	0.93	0.67	1.30	0.679	1.02	0.68	1.53	0.930	1.53	1.00	2.36	0.052
Levels 5–7	1.28	0.93	1.75	0.124	1.39	0.92	2.09	0.121	0.84	0.39	1.78	0.644	1.37	0.65	2.86	0.408
Cancer stage																
Stage I	1.00				1.00				1.00				1.00			
Stage II	2.23	1.58	3.13	<0.001	1.83	1.16	2.90	<0.001	3.68	1.65	8.22	0.001	1.97	0.98	3.98	0.058
Stage III	5.07	3.62	7.08	<0.001	3.92	2.50	6.14	<0.001	8.60	3.87	19.11	<0.001	5.68	2.87	11.22	<0.001
Stage IV	25.82	18.46	36.10	<0.001	21.09	13.35	33.31	<0.001	42.51	19.11	94.55	<0.001	32.90	16.69	64.84	<0.001
Treatment																
Surgery	1.00				1.00				1.00				1.00			
Chemotherapy	1.29	1.01	1.65	0.045	1.06	0.74	1.53	0.740	2.07	1.28	3.36	0.003	1.18	0.70	2.01	0.535
Radiotherapy	3.22	1.93	5.37	<0.001	3.43	1.82	6.46	<0.001	1.29	0.30	5.61	0.730	16.55	4.77	57.46	<0.001
Surgery and chemotherapy	0.79	0.64	0.96	0.021	0.64	0.48	0.86	0.003	0.90	0.59	1.36	0.608	0.84	0.55	1.30	0.440
Surgery and radiotherapy	1.78	1.16	2.72	0.008	2.13	1.25	3.61	0.005	2.69	1.04	6.92	0.040	0.65	0.21	1.99	0.447
Chemotherapy and radiotherapy	1.46	1.10	1.95	0.009	0.87	0.55	1.36	0.538	2.35	1.36	4.04	0.002	1.75	0.98	3.12	0.060
Surgery, chemotherapy, and radiotherapy	1.07	0.86	1.33	0.530	0.79	0.57	1.09	0.155	1.48	0.97	2.28	0.071	1.14	0.73	1.78	0.560
Hospital ownership																
Public	1.00				1.00				1.00				1.00			
Private	0.98	0.87	1.11	0.783	1.07	0.88	1.29	0.499	0.82	0.66	1.03	0.084	0.91	0.72	1.15	0.449
Hospital level																
Medical center	1.00				1.00				1.00				1.00			
Regional hospital	1.17	1.03	1.32	0.014	1.27	1.04	1.55	0.019	0.88	0.70	1.09	0.247	1.23	0.68	2.25	0.495
District hospital	1.08	0.79	1.48	0.621	1.10	0.71	1.70	0.685	1.12	0.56	2.22	0.749	1.38	1.09	1.74	0.007

^a^ Adjusted for monthly salary, education, gender, age, marital status, CCI score, urbanization level, cancer stage, treatment, hospital ownership, and hospital level; ^b^ Cox proportional hazards model; ^c^ adjusted for education, gender, age, marital status, CCI score, urbanization level, cancer stage, treatment, hospital ownership, and hospital level.

**Table 6:** Socioeconomic status associated with death risk of colorectal cancer patients stratified by age.

Variables	Age 40–54				Age 55–64				Age ≥65			
	aHR ^a^	95% CI		*p*-Value ^b^	aHR ^a^	95% CI		*p*-Value ^b^	aHR ^a^	95% CI		*p*-Value ^b^
Monthly salary												
≤Q1	1.16	0.75	1.80	0.511	1.09	0.76	1.54	0.649	1.14	0.94	1.39	0.177
Q1–Q3	0.99	0.69	1.41	0.955	0.72	0.52	1.00	0.050	0.88	0.70	1.10	0.262
>Q3	1.00				1.00				1.00			
Education												
Elementary school and under	1.85	1.02	3.37	0.044	1.72	1.05	2.81	0.031	1.29	0.91	1.83	0.151
Junior high school	2.06	1.19	3.56	0.010	1.30	0.76	2.23	0.346	0.98	0.66	1.46	0.917
Senior high/vocational school	1.52	0.94	2.46	0.088	1.25	0.78	2.00	0.363	1.03	0.71	1.49	0.876
Junior college/university and above	1.00				1.00				1.00			

^a^ Adjusted for gender, age, marital status, CCI score, urbanization level, cancer stage, treatment, hospital ownership, and hospital level; ^b^ Cox proportional hazards model.

## Data Availability

Data are available from the Health and Welfare Data Science Center of the Ministry of Health and Welfare (MOHW), Taiwan. All interested researchers can apply for using the database managed by the MOHW. Due to legal restrictions imposed by the Taiwanese government related to the Personal Information Protection Act, the database cannot be made publicly available. Any raw data are not allowed to be brought out from the Health and Welfare Data Science Center. The restrictions prohibited the authors from making the minimal data set publicly available.
